# Extracellular vesicle-associated procoagulant phospholipid and tissue factor activity in multiple myeloma

**DOI:** 10.1371/journal.pone.0210835

**Published:** 2019-01-14

**Authors:** Thøger Nielsen, Søren Risom Kristensen, Henrik Gregersen, Elena Manuela Teodorescu, Gunna Christiansen, Shona Pedersen

**Affiliations:** 1 Department of Clinical Biochemistry, Aalborg University Hospital, Aalborg, Denmark; 2 Department of Clinical Medicine, Aalborg University, Aalborg, Denmark; 3 Faculty of Medicine, Aalborg University Hospital, Aalborg, Denmark; 4 Department of Haematology, Aalborg University Hospital, Aalborg, Denmark; 5 Department of Biomedicine, Aarhus University, Aarhus, Denmark; Universita degli Studi di Torino, ITALY

## Abstract

Multiple myeloma (MM) patients have increased risk of developing venous thromboembolism, but the underlying mechanisms and the effect on the coagulation system of the disease and the current cancer therapies are not known. It is possible that cancer-associated extracellular vesicles (EV), carrying tissue factor (TF) and procoagulant phospholipids (PPL) may play a role in thrombogenesis. The aim of this study was to perform an in-depth analysis of procoagulant activity of small and large EVs isolated from 20 MM patients at diagnosis and after receiving first-line treatment compared with 20 healthy control subjects. Differential ultracentrifugation at 20,000 × *g* and 100,000 × *g* were used to isolate EVs for quantitative and phenotypical analysis through nanoparticle tracking analysis, Western blotting and transmission electron microscopy. The isolated EVs were analyzed for procoagulant activity using the calibrated automated thrombogram technique, a factor Xa-based activity assay, and the STA Procoag-PPL assay. In general, MM patients contained more EVs, and immunoelectron microscopy confirmed the presence of CD9- and CD38-positive EVs. EVs in the 20,000 × *g* pellets from MM patients exerted procoagulant activity visualized by increased thrombin generation and both TF and PPL activity. This effect diminished during treatment, with the most prominent effect observed in the high-dose chemotherapy eligible patients after induction therapy with bortezomib, cyclophosphamide, and dexamethasone. In conclusion, the EVs in patients with MM carrying TF and PPL are thus capable of exerting procoagulant activity.

## Introduction

Cancer patients have a 4–7-fold higher risk of venous thromboembolism (VTE) than does the general population, but the risk in different cancer types varies, and the frequency of VTE in cancer patients is between 1–8% [1–3]. Patients with multiple myeloma (MM) have a considerably increased risk of VTE, partly because the associated treatment may be thrombogenic [4–6]. Although several factors, such as age, acquired protein C resistance, coagulation factor VIII, von Willebrand factor, and interleukin-6, have been proposed as contributors to this hypercoagulable state, the mechanisms causing VTE in patients with MM are not clearly understood [7–9]. A possible contributing factor is an increased level of tissue factor (TF), a central coagulation factor in initiating haemostasis that triggers thrombin generation [10]. It has been reported that aberrant TF expression is linked to cancer pathophysiology, e.g., angiogenesis [11]. Anionic procoagulant phospholipids (PPL), such as phosphatidylserine, act as important cofactors necessary for the formation of coagulation complexes but have also been proposed to be involved in cancer pathogenesis [12]. TF and PPL can be present in plasma in circulating extracellular vesicles (EV) with procoagulant properties. In malignancy, EVs from the cancer cells are involved in several pleiotropic processes, such as metastasis, angiogenesis, and immunomodulation [13,14]. Because they may also carry TF and PPL, likely on the large EVs, so-called microvesicles (MV), these EVs may play a significant role in haemostasis and VTE-risk in various diseases, including MM [15–18]. Auwerda et al [16] reported a microparticle-associated TF-activity in MM patients receiving high-dose chemotherapy (HDCT). The aim of this study was to investigate the procoagulant effect of EVs from patients with newly diagnosed MM compared with controls, hypothesizing that EVs in patients with MM are procoagulant.

## Materials and methods

### Study population

A total of 20 newly diagnosed patients with MM according to the International Myeloma Working Group criteria, were included in the study at the Department of Haematology, Aalborg University Hospital, Denmark. At inclusion, none of the patients received anti-coagulation therapy and had no history of previous VTE or other malignancies. The patients were staged according to the International Staging System (ISS) for multiple myeloma. Patients eligible for HDCT received three or four series of bortezomib, cyclophosphamide, and dexamethasone (VCD) induction therapy, and then after leukapheresis proceeded to high-dose melphalan with stem cell support. This patient group will be referred to as the VCD induction therapy group. The patients ineligible for HDCT received a conventional treatment consisting of melphalan, prednisone, and bortezomib (MPV) and will henceforth be referred to as the conventional treatment group. Treatment response was assessed through the multiple myeloma treatment response criteria described by the International Myeloma Work Group and as a relative reduction in M-protein post treatment. Plasma samples from 20 healthy partly matched subjects were collected as controls. The study was conducted in agreement with the Declaration of Helsinki and approved by the ethical committee of Northern Jutland (N-20130075). Written informed consent was acquired from all participants at inclusion in the study.

### Sample collection and EV isolation

Samples were collected from the patients at diagnosis and after their first-line anti-myeloma treatment, i.e. approximately four weeks after VCD induction therapy (prior to stem cell transplantation) or MPV treatment dependent on treatment regimen. Venous blood was collected in 6-mL 0.105 M (3.2%) trisodium citrate tubes (Becton Dickinson, Franklin Lakes, NJ, USA). Within one hour after collection, platelet-free plasma (PFP) was extracted by a double centrifugation at 2,500 × *g* at room temperature for 15 minutes according to international recommendations [19,20]. Plasma collection was stopped one cm from the buffy coat and the pellet in the consecutive spin. The PFP was stored at -80°C until analysis. The isolation process of the EVs consisted of a two-step ultracentrifugation in an Avanti J-30i equipped with a JA-30.50 rotor, k-factor 280 (Beckman Coulter, Brea, CA, USA). The first batch of EVs was pelleted from 1 ml PFP by centrifugation at 20,000 × *g* (20K) for 30 minutes at 4°C. The 20K pellets were washed once in 1 ml phosphate-buffered saline (PBS) at the same *g*-force and duration. Residual EVs were pelleted by centrifugation of the supernatant at 100,000 × *g* (100K) for 60 minutes at 4°C. Likewise, the 100K pellets were washed once in 1 ml PBS at the same *g*-force and duration. To create an equal baseline in the coagulation analyses for the different patients and controls, all pellets were finally resuspended in standard pool plasma (SPP). The pellets were resuspended in 200 μl SPP (i.e., they were five times more concentrated). SPP was collected from a single donor analogous to the PFP extraction described above. For the quantitative and phenotypical analyses, pelleted EVs were resuspended in 200 μl PBS.

### Nanoparticle tracking analysis

Nanoparticle tracking analysis was applied to determine the size and concentration of particles in the pellets and confirm that their size was equivalent to that of EVs. Particles were tracked on a LM10-HS system with a 405 nm laser (Malvern Instruments, Malvern, UK) and visualized with a Luca-DL EMCCD camera (Andor Technology, Belfast, UK). The 0.1 μm standard silica beads were used to calibrate the analysis settings. Settings applied were camera level 10 and detection threshold 2 with blur 9×9. A total of five videos of 30 seconds each was recorded for the individual samples. Prior to analysis, the samples were diluted in PBS to ensure a particles per frame count within the manufacturer’s recommendations. Particles were tracked, quantified, and size enumerated using the Nanosight NTA software version 3.0 (Malvern Instruments).

### Western blotting

Western blotting was performed to identify EVs positive for the commonly used EV-marker CD9 and the therapeutic target marker CD38 expressed abundantly on myeloma cells. The pellet pools were lysed with 2 × Laemmli Sample Buffer (Bio-Rad Laboratories, Hercules, CA, USA), boiled for 5 minutes at 95°C, and separated in MiniProtean TGX 4–15% gels (Bio-Rad Laboratories). The proteins were transferred to Amersham Hybond P 0.20 PVDF blotting membranes (clone M-L13, GE Healthcare, Little Chalfont, UK) for 60 minutes at 100 V and subsequently blocked in 5% (w/v) skim milk blocking buffer for 60 minutes. The membranes were incubated with primary monoclonal mouse anti-CD9 antibody (clone M-L13, BD Pharmingen, San Diego, CA, USA) and monoclonal human anti-CD38 antibody (daratumumab; Jannsen-Cilag A/S, Birkeroed, Denmark) diluted 1:1000 with blocking buffer. Secondary antibodies used were horseradish peroxidase-conjugated polyclonal goat anti-mouse antibodies (Dako, Glostrup, Denmark) and polyclonal goat anti-human antibodies (Abcam, Cambridge, UK). Detection of membranes was performed using ECL Prime Western Blotting detection reagent (GE Healthcare) and the PXi 4 system with the GeneSys software version 1.5.4.0 (Syngene, Cambridge, UK). The bands were quantified with ImageJ 1.50e software (NIH, Bethesda, MD, USA).

### Transmission electron microscopy and immunogold labelling

To detect vesicles that structurally resembled EVs in the pellets, transmission electron microscopy was performed. The procedure used was in accordance with previous studies [21,22] with minor modifications. Five microliter pooled pellet suspension was mounted on a carbon-coated, glow discharged 400 mesh Ni grid (SPI supplies, Chester, PA, USA) for 30 seconds, followed by staining with one drop of 1% (w/v) phosphotungstic acid (Ted Pella, Caspilor AB, Lindingö, Sweden) pH 7.0. Then, the grid was blotted dry on filter paper. Detection of EV subpopulations was achieved through transmission electron microscopy with immunogold labelling. Samples were mounted on carbon-coated, glow discharged 400 mesh Ni grids for 30 seconds and washed three times with PBS. Grids were blocked with 0.5% ovalbumin (Sigma-Aldrich, St. Louis, MO, USA) in PBS and then incubated with primary monoclonal mouse anti-CD9 antibody (clone M-L13, BD Biosciences, Albertslund, Denmark) or anti-CD38 antibody (daratumumab; Jannsen-Cilag A/S) 1:50 in 0.5% ovalbumin in PBS for 30 minutes at 37°C. After three washes in PBS, the grids were incubated with 10 nm gold-conjugated goat anti-mouse secondary antibody (British BioCell, Cardiff, UK) diluted 1:25 in 0.5% ovalbumin in PBS in advance. The grids were then washed with three drops of PBS and incubated on three drops of 1% cold fish gelatin (Sigma-Aldrich) for 10 minutes per drop. Subsequently, the grids were washed with three drops of PBS and stained with one drop of 1% (w/v) phosphotungstic acid at pH 7.0. The grids were then blotted dry. To visualize the samples, a JEM-1010 transmission electron microscope (JEOL, Tokyo, Japan) operated at 60 keV was used. An electron-sensitive CCD camera (KeenView, Olympus, Tokyo, Japan) was used to capture images and a grid-size replica (2,160 lines/mm) and the ImageJ 1.50r software (NIH, Bethesda, MD, USA) was used to assess size of visualized EVs.

### Thrombin generation assay (calibrated automated thrombogram)

Thrombin generation was assessed according to the protocol for the calibrated automated thrombogram (CAT) previously described by Hemker et al [23]. The 80 μL EV suspension was mixed with 20 μL PRP reagent (Thrombinoscope B.V., Maastricht, the Netherlands) containing 1 pM TF and no phospholipids. Coagulation was initiated by addition of 20 μL FluCa buffer containing CaCl_2_ and fluorogenic substrate (FluCa kit, Thrombinoscope B.V.). The reaction was measured in an automated Fluoroscan Ascent (Thermo Scientific, Waltham, MA, USA) and peak height, lag time, time-to-peak, and velocity index were calculated using the Thrombinoscope software version 5.0 (Thrombinoscope B.V.). Endogenous thrombin potential (ETP, area under the curve) was calculated manually and for the whole test duration of 60 minutes. SPP with buffer (blank, i.e., no addition of EVs) was measured several times to establish a reference range for the SPP on each parameter.

### Procoagulant phospholipid activity assay

The STA-Procoag-PPL assay (Diagnostica Stago, Asnieres, France) was used to measure the activity of EV-associated PPL. In this assay, all of the coagulation factors were supplied at physiological levels by PPL-depleted plasma, apart from PPL, which was provided by EVs in the pellets. The 25 μL EV suspension was diluted in 25 μL Owren-Koller buffer. The reaction was triggered by Ca^2+^ and factor Xa (FXa). The assay measures a clotting time (seconds), which is inversely proportional to PPL activity, meaning a shorter clotting time indicates an increased PPL activity. The assay was conducted on a STA-Compact (Diagnostica Stago) in accordance with the manufacturer’s protocol. SPP with buffer only (blank) was measured several times to establish a reference range for the PPL clotting time.

### MV-TF activity assay

MV-TF activity and MV-FXa generation was measured with an adapted method from Wang et al [24]. First, 600 μL plasma was diluted in 1 mL HBSA buffer (137 mM NaCl, 5.38 mM KCl, 5.55 mM glucose, 10 mM HEPES, 0.1% (w/v) bovine serum albumin, pH 7.4) and centrifuged at 20,000 × *g* for 15 minutes at 4°C in order to pellet microvesicles. The pellets were washed once in 1 mL HBSA and resuspended in 180 μL HBSA. The samples were then incubated with monoclonal mouse anti-CD142 antibody (clone HTF-1, BD Pharmingen) or control IgG from mouse serum (Sigma-Aldrich) for 15 minutes at room temperature in a 96-well plate. After incubation, 50 μL HBSA containing 10 mM CaCl_2,_ 73 nM FX (Enzyme Research Laboratories, South Bend, IN, USA), and 2.4 nM factor VIIa (Enzyme Research Laboratories) was added to each sample and incubated for two hours at 37°C. The reaction was stopped by addition of 25 μL HBSA containing 25 mM EDTA. Then, 25 μL of 4 mM chromogenic Pefachrome FXa 8595 (Pentapharm, Basel, Switzerland) was added to the wells and incubated at 37°C for 15 minutes. The plate was read at absorbance 405 nm on a Fluostar Optima (BMG Labtech, Ortenberg, Germany). Innovin (Siemens Healthcare, Erlangen, Germany) was used to generate a standard curve to calculate the procoagulant activity of microvesicles.

### Statistical analysis

The results are expressed by the means ± standard deviation or as boxplots depicting median, the 25 and 75 percentiles and whiskers min to max. Differences between the two groups and pellets were determined with either Student’s *t-*test or the Mann-Whitney U test depending on the distribution type. The Pearson correlation coefficient was used to signify associations between variables. Differences before and after treatment of the MM patients were determined using either paired *t-*tests or the Wilcoxon matched-pairs signed rank test. P-values less than 0.05 were considered statistically significant. Statistical analyses were performed using IMB SPSS Statistics version 24 (SPSS, Chicago, IL, USA) and Graph Pad Prism version 6 (GraphPad Software, La Jolla, CA, USA).

## Results

### Patient characteristics

A total of 20 patients with a median age of 72 years (range 40–84) and a male/female distribution of 11/9 were included in this study. Two patients (10%) had stage I, seven (35%) had stage II, and 11 had (55%) stage III disease according to the ISS for multiple myeloma. None of the patients received anti-coagulant prophylaxis at inclusion or during the period they were followed until the collection of the second sample. MM patient characteristics at diagnosis are summarized in Table 1, showing expected abnormalities in some of the patients such as low haemoglobin, slightly increased creatinine, acute phase reaction (increased C-reactive protein, fibrinogen and FVIII) and positive D-dimer. The controls had a median age of 64 years (range 56–67) and a male/female distribution of 11/9. They were all healthy with no biochemical abnormalities. From 16 of the 20 patients, a post-treatment sample was obtained, whereas the remaining four patients died (2–4 months after the initial sample). Five of the 16 patients received the VCD induction therapy. For the 11 remaining patients in the conventional treatment group, 10 received MPV and one was treated with lenalidomide and dexamethasone. Four patients died before a follow-up sample was collected–three from sepsis and one of unknown reasons. Demographic characteristics and treatment response before and after first-line treatment for both treatment groups are listed in Table 2 and additional patient characteristics are listed in the supplemental information (S1 Table).

**Table 1:** Characteristics of the multiple myeloma patients at diagnosis.

* *	Multiple myeloma	*Reference range* *(male / female)*
Number of patients	20	
Age, years	70 ± 10	
Male percentage	55%	
ISS stage		
I	2 (10%)	
II	7 (35%)	
III	11 (55%)	
M-protein, g/L	41.5 ± 19.9	
IgG, *n*	14 (70%)	
kappa	*11 (55%)*	
lambda	*3 (15%)*	
IgA, *n*	6 (30%)	
kappa	*4 (20%)*	
lambda	*2 (10%)*	
INR	1.1 ± 0.2	*<1*.*3*
APTT, s	30 ± 4	*25–40*
Fibrinogen, μmol/L	9.4 ± 3.3	*5*.*0–12*.*0*
D-dimer, mg/L	0.80 ± 0.27	*<0*.*30*
Antithrombin, ×E9 IU/L	0.88 ± 0.17	*0*.*80–1*.*20*
Factor VIII, U/mL	1.60 ± 0.73	*0*.*60–1*.*60*
Protein C, U/mL	1.10 ± 0.39	*0*.*70–1*.*40*
Creatinine, μmol/L	89 ± 23 / 77 ± 23	*60–105 /* *45–90*
Carbamide, mmol/L	7.0 ± 2.6 / 6.2 ± 1.2	*3*.*5–8*.*1 /* *3*.*1–7*.*9*
Pt-estimated GFR, mL/min	74 ± 17	*>60*
κ-chain, free, mg/L	1153.1 ± 3723.9	*3*.*3–19*.*4*
λ-chain, free, mg/L	299.8 ± 697.1	*5*.*7–26*.*3*
Calcium, mmol/L	2.48 ± 0.15	*2*.*20–2*.*55*
CRP, mg/L	7.5 ± 22.7	*<8*.*0*
Albumin, g/L	30 ± 4	*34–45*
Protein, g/L	106 ± 18	*62–78*
ALAT, U/L	23 ± 10	*10–50*
Haemoglobin, mmol/L	6.7 ± 1.5 / 6.0 ± 0.6	*8*.*3–10*.*5 /* 7.3–9.5
Erythrocytes, ×E12/L	3.44 ± 0.80 / 3.18 ± 0.38	*4*.*30–5*.*70 /* *3*.*90–5*.*20*
Platelets, ×E9/L	198 ± 57 / 248 ± 52	*145–350 /* *165–400*
Leukocytes, ×E9/L	6.3 ± 2.2	*3*.*5–10*.*0*

ISS = international staging system; IgG = immunoglobulin G; IgA = immunoglobulin A; INR = international normalized ratio; APTT = activated partial thromboplastin time; GFR = glomerular filtration rate; CRP = C-reactive protein; ALAT = alanine transaminase.

**Table 2:** Demographic characteristics of patients the conventional or induction therapy groups including treatment response.

	Conventional therapy	VCD induction therapy
Number of patients	11	5
Age, years*	76 ± 5	64 ± 5
Male gender	55%	40%
*ISS stage*		
I	1 (9%)	0 (0%)
II	6 (55%)	2 (40%)
III	4 (36%)	3 (60%)
*Treatment*		
VCD	0 (0%)	5 (100%)
MPV	10 (91%)	0 (0%)
LEN-DEX	1 (9%)	0 (0%)
*Treatment response*		
Very good partial response	3 (28%)	4 (80%)
Partial response	4 (36%)	1 (20%)
Stable disease	4 (36%)	0 (0%)
M-protein posttreatment reduction, %	58 ± 25	90 ± 9

*Mean ± standard deviation; LEN-DEX = lenalidomide and dexamethasone.

### Isolation and characterization of EVs

**Fig 1:**
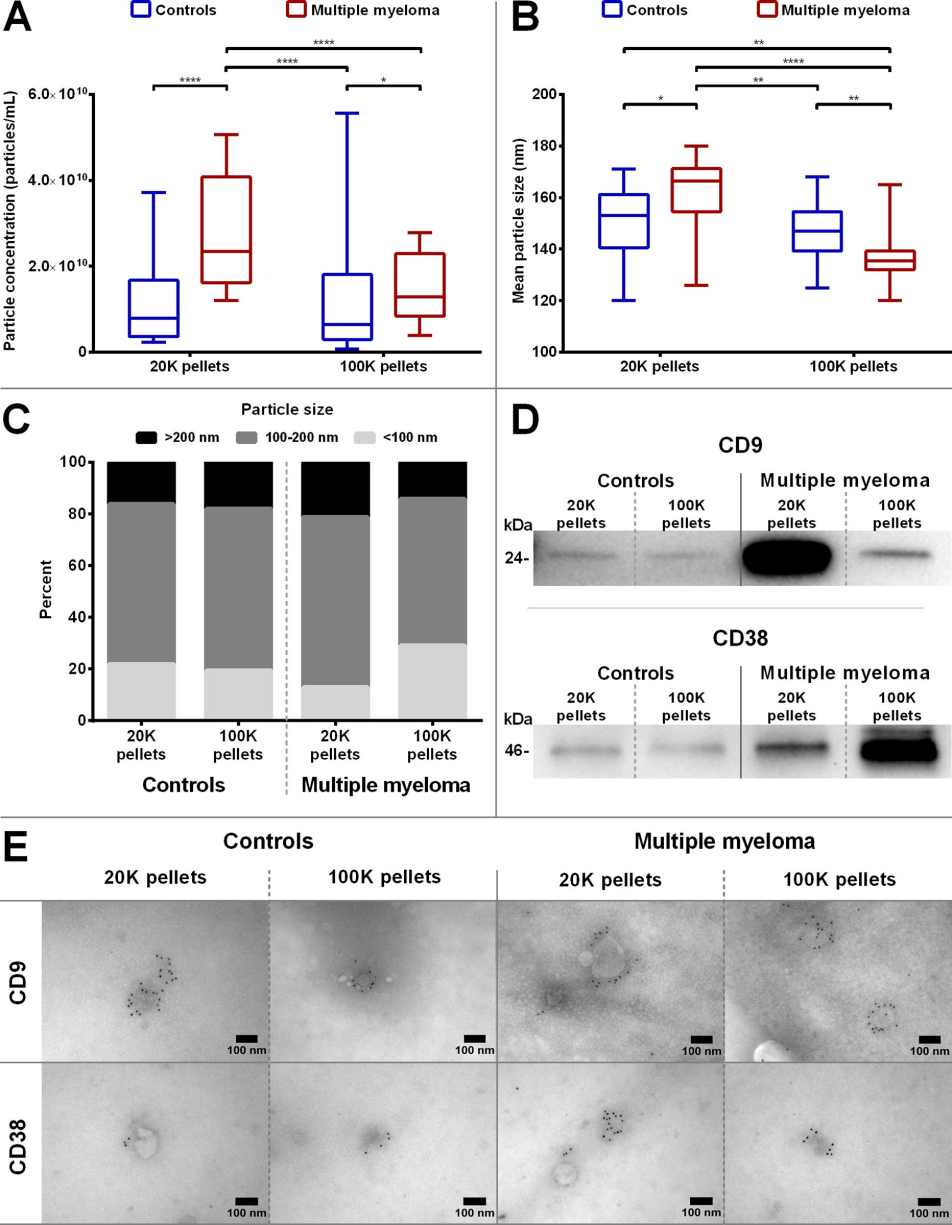
Analysis of EV characteristics. Nanoparticle tracking analysis was performed on each pellet (20K and 100K) for controls and MM patients to determine *A*) particle concentrations and *B*) mean particle size. The boxplots depict the median, the 25 and 75 percentiles and the whiskers min to max. *P<0.05; **P<0.01; ****P<0.0001. *C*) The distribution of particle sizes was grouped into three subgroups (<100 nm, 100–200 nm, and >200 nm). *D*) The pellet pools were analysed by Western blotting for EV-marker CD9 and ectoenzyme CD38. Equivalent volumes of each pellet pool (20K and 100K) from both controls and MM were loaded on the gels. As expected, tetraspanin CD9 was present in all pellet types but enriched in MM pellets, especially in the 20K pellet pool. CD38 was found in all pellet pools, but most abundant in MM pellets (mostly in the 100K pellet pool). *E*) Immunoelectron microscopy images of gold immunolabelled CD9^+^ and CD38^+^ EVs in pellet pools of control and MM pellets (20K and 100K pellets). Images include scale bars determined with ImageJ software.

In general, significantly more particles (P < 0.01) were isolated in the MM pellets than in the control pellets, with the majority of particles isolated from MM patients in the 20K pellet (Fig 1A). In both groups, 20K pellets showed the largest mean particle size (P < 0.01) compared to the 100K pellets, although only a minor difference was observed for the control pellets (Fig 1B). The 20K pellets in the MM group had the highest percentage (86%) of particles larger than 100 nm in size (Fig 1C). Both control and MM pellets contained CD9^+^ EVs, but more CD9^+^ EVs were present in the MM pellets (more than a 3-fold increase), with the most pronounced signal in the 20K pellets (6-fold) in contrast to the pooled control pellets (Fig 1D). CD38^+^ EVs were present in MM 20K and 100K pellets, with the most distinct band present in the latter. Both 20K and 100K pooled pellets from the controls displayed faint CD38 bands, and 20K and 100K pellets of MM were 3 to 6-fold stronger, respectively. Immunoelectron microscopy showed that some EVs isolated from both control and MM pellets were CD9^+^ and CD38^+^ (Fig 1E).

### Procoagulant analysis of EVs

**Fig 2:**
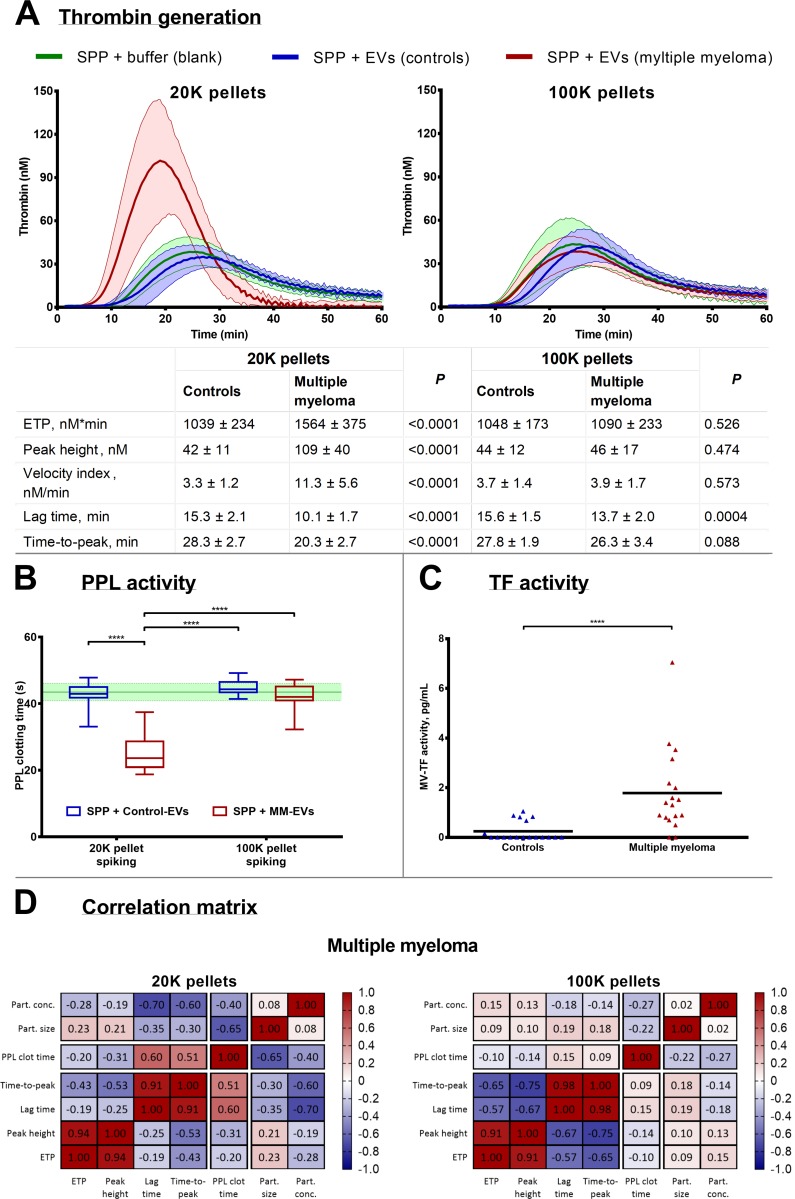
Analysis of procoagulant activity of EVs in SPP. *A*) Thrombograms (mean ± standard deviation) depicting thrombin generation when SPP is ‘spiked’ with isolated EVs from controls and MM patients. The results on individual thrombin generation parameters (ETP, peak height, velocity index, lag time, and time-to-peak) are listed in the table as the means ± standard deviation including *P*. *B*) PPL activity measured in clotting time differences in SPP ‘spiked’ with isolated EVs. The boxplots depict the median, the 25 and 75 percentiles and the whiskers min to max and the green line and area represent the reference range (mean ± standard deviation) of the SPP. *C*) Analysis of MV-associated TF was performed on MV suspensions, and MM patients contained overall more TF than controls. *D*) Correlation matrix depicting the Pearson’s r for correlations between coagulation and particle analyses for the MM pellets. ****P<0.0001.

The isolated EVs were resuspended in SPP and analysed for procoagulant activity. EVs in the 20K pellets from MM patients resulted in significantly increased peak height (>1.8-fold, P < 0.0001), velocity index (2.7-fold, P < 0.0001), and ETP (60%, P < 0.0001) compared to the baseline values of the SPP (Fig 2A). Lag time and time-to-peak were both shortened significantly (P < 0.0001) in the MM 20K pellets. In addition, the procoagulant phospholipid activity for EVs in the MM 20K pellets showed significantly reduced PPL clotting time (P < 0.0001), whereas the MM 100K together with the control 20K and 100K pellets revealed no changes in thrombin generation and PPL activity (Fig 2B). MVs in MM patients contained more TF activity (P < 0.05) than those of the controls (Fig 2C).

In general, a profound difference was observed between EVs in 20K and 100K pellets, with the former being the most procoagulant. The increased PPL activity of 20K EVs from MM patients correlated with the shortened lag time and time-to-peak from thrombin generation (P < 0.01 and P < 0.05, respectively). A clear tendency was present in the correlation between ETP and peak height. Furthermore, the elevated PPL activity of EVs from MM patients correlated to the mean size of the larger particles (i.e., the mean EV size, P < 0.01), as seen in the correlation matrix in Fig 2D. The individual correlations are displayed in supplemental information (S1 Fig).

### Treatment of multiple myeloma–possible implications for EVs

**Fig 3:**
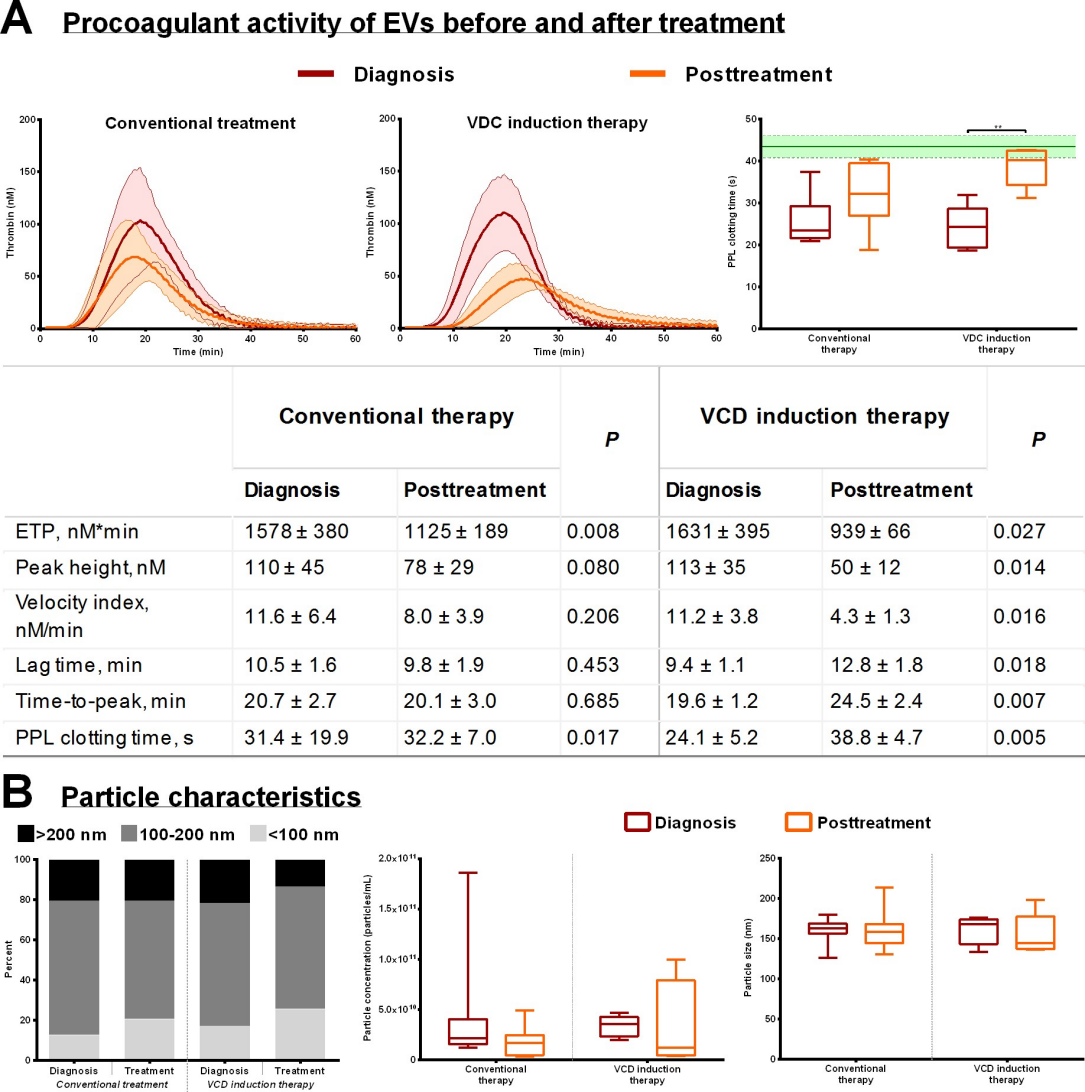
The procoagulant activity of EVs from 20K pellets of MM patients at diagnosis and after the first-line treatment regimen. *A*) (*Left*, *middle*) Thrombograms (mean ± standard deviation) depicting the outcome of EV-mediated thrombin generation before and after conventional therapy (HDCT ineligible patients) or a VCD induction therapy (HDCT eligible patients). The results on individual thrombin generation parameters are listed in the table as the means ± standard deviation including *P*. (*Right*) The effect of treatment on PPL activity of EVs ‘spiked’ into SPP, **P<0.01. The green line and area represent the reference range (mean ± SD) of the SPP. *B*) Size distribution (*left*), particle concentration (*middle*), and mean size (*left*) before and after first-line treatment as measured by the means of nanoparticle tracking analysis. All boxplots depict the median, the 25 and 75 percentiles and the whiskers min to max.

Analysis of procoagulant activity of EVs in 20K pellets on thrombin generation after the patients were treated with or without VCD mostly showed a reduced coagulation activity, but this was most notable in the patients receiving VCD (Fig 3A). The mean ETP was reduced by more than 42% (P < 0.05) after treatment in patients receiving VCD induction therapy, whereas the mean ETP for those receiving conventional treatment was reduced by 29% (P < 0.01). The mean peak height of the VCD induction group was reduced by more than 50% (P < 0.05) compared to a non-significant reduction of 30% in patients in the conventional treatment group. Moreover, in the VCD group, lag time was increased by 25% (P < 0.05) and time-to-peak by more than 37% (P < 0.01), and these two measures were almost unchanged for the patients treated conventionally. A similar tendency was observed in PPL activity, with a significant median increase in PPL clotting time of 15.9 seconds (65%, P = 0.063) in the VCD group after treatment compared to 7.8 seconds (33%, P = 0.175) for patients in the conventional treatment group. Fig 3B shows that there was a small decrease in particle quantity and the mean particle size after treatment in both groups but the differences were not significant. The distribution between small and large EVs were not changed much in the conventional treatment group whereas in the VCD group the fraction with the largest particles (>200 nm) diminished by almost 50% (Fig 3B). Graphic illustrations depicting the effect of treatment on each thrombin generation parameter and PPL clotting time for the two treatment regimens, including the individual patients, are listed in the supplemental information (S2 Fig).

## Discussion

This setup to investigate procoagulant activity demonstrated a substantially higher thrombin generation and both TF and PPL activity in EVs in patients with MM than in healthy control subjects. This increase in procoagulant activity, however, diminished markedly in the patients receiving VCD induction therapy and to a lesser extent in those that received the conventional treatment. These results indicate that the procoagulant activity in MM can be ascribed to the larger EVs, which likely exert their procoagulant activity through PPL and TF. Furthermore, we demonstrated that some of the EVs possibly originate from the cancerous B cells.

EVs secreted by cancer cells have a function promoting their survival, angiogenesis, and immune escape, and therefore, circulating EVs may be present in higher quantities in cases of malignancy [25,26]. In the present study, we isolated EVs through differential ultracentrifugation and detected increased levels of EVs of various sizes in patients with MM (Fig 1A–1C). EVs in both patients and controls were positive for CD9 (Fig 1D and 1E), a marker frequently used for common EVs [27,28]. Most of the CD9^+^ vesicles were discovered in the 20K pellet, which also contained the largest fraction of EV > 100 nm. Moreover, MM patients contained markedly more CD38^+^ EVs than did the controls. The elevated expression of CD38 indicates that a substantial fraction of the EVs found in MM are linked to the malignancy, as EVs released by MM cells are known to be enriched in CD38 [29]. Contrary to the CD9 expression, our data suggest that the majority of CD38 are expressed by smaller EVs, since the 100K pellet contained fewer EVs and a larger fraction of EVs were <100 nm compared to the 20K pellet.

We aimed to investigate EVs in MM patients and their potential procoagulant effect on the haemostatic system, which has been demonstrated in other cancers [30,31]. To analyse the EV-mediated procoagulant activity, we used a model recently described [32], in which differential ultracentrifugation was applied on plasma samples. The CAT method, being a global coagulation test, provides information of the entire system including TF and PPL activity [33–36]. Presence of TF will primarily shorten lag time and time-to-peak, whereas a high PPL activity will increase ETP and peak height [35]. The method has been used to establish thrombin generation as a predictive marker for VTE in MM patients [37,38]. The STA-Procoag-PPL kit specifically measures the PPL activity that may be exerted by EVs. Other studies have demonstrated the effect of PPL-exposing EVs exerting procoagulant activity in different pathological conditions, such as cancer [39–41]. Finally, we performed a FVIIa dependent FXa generation assay on the pellets to detect TF activity. Several modifications of this technique has been used in many other cases to detect TF activity of the larger EVs in relation to VTE occurrence [15,42,43]. This test has also been used to detect elevated TF activity in patients with MM [16,44].

In the present study, we found that patients with MM do contain procoagulant EVs that increase the amount of thrombin generated, as demonstrated by the CAT method. Furthermore, both TF and PPL activity were also increased. The procoagulant EVs are probably the larger EVs since the 20K pellets profoundly reduced both lag time and time-to-peak (Fig 2A), indicating that some EVs in MM patients carry TF embedded in their membrane, which is in accordance with the specific measurements of increased TF activity compared to almost none in the control group (Fig 2C). Furthermore, PPL activity in MM patients is higher in the 20K pellets (Fig 2B), in accordance with increased peak height and velocity index (Fig 2A). Additionally, increased PPL activity in larger EVs correlated with shorter lag time and time-to-peak in the CAT analysis, thus suggesting an association between PPL and TF. Peak height and ETP also showed a similar trend of PPL dependency with higher peak height and ETP with more PPL activity. Both the quantity and size of the large EVs are likely of importance for the procoagulant potency of the EVs, but the overall trend is that the 20K EVs are definitely more procoagulant compared to EVs in the 100K pellets. Since the CD38 positive EVs (which probably are derived from cancer cells) were mainly present in the 100K pellet the procoagulant effect of EVs do not seem to be closely associated to this fraction, but we cannot from this investigation resolve whether the procoagulant EVs are derived from cancer cells or other cells.

The procoagulant activity of EVs from the MM patients diminished after treatment; however, patients treated with induction therapy had the most distinct effect (Fig 3A), eliminating the majority of the procoagulant activity of the EVs. This result may be due to reduction of the amount of particles >200 nm, the supposed MVs, which was reduced considerably after treatment compared to those being treated conventionally (Fig 3B). An important feature to mention is that the patients in the VCD induction therapy group respond better overall to their treatment (Table 2), which may thus impact the reduced procoagulant activity of EVs we observe. The reduction in both lag time and time-to-peak in the VCD induction therapy group indicates reduced TF activity, supported by others reporting decreasing TF activity in MM patients receiving induction chemotherapy [16]. In contrast, Leiba et al [37] reported no difference in thrombin generation in plasma from MM patients after HDCT.

The study is limited by the small sample size, especially after being divided into two treatment groups depending whether or not the patients were eligible for HDCT. Nevertheless, the differences between the MM patents and the controls were quite large and significant. The samples were collected over a period of one and a half year, which may have a minor impact on EV quantity and size distribution, however, the sample collection was uniformly conducted between patients and controls. Furthermore, no VTE events occurred in any group; therefore, we are unable to link procoagulant EVs in MM to an increased VTE risk. There was a minor difference in the mean age between controls and patients, partly because it was difficult to recruit elderly controls. However, the difference is minimal and probably also of minor importance.

In conclusion, we found that patients newly diagnosed with MM contain more and larger EVs in their plasma and that these EVs exert procoagulant activity, resulting in an increased thrombin generation and TF and PPL activity. This EV-mediated procoagulant effect diminishes after the initiation of treatment, especially in patients receiving VCD induction therapy. This finding may explain, at least in part, why MM patients have an increased risk of VTE; however, this warrants confirmation in larger cohorts where the effect of administration of a more thrombogenic anti-myeloma treatment also could be addressed.

## Supporting information

S1 Fig Correlations between coagulation assays and nanoparticle tracking analysis performed on EVs from patients with MM. P-values or non-significant (NS) correlations are depicted in the corresponding colour for 20K or 100K pellets.
(DOCX)

S2 Fig The effect of treatment on procoagulant EVs in 20K pellets from MM patients eligible for HDCT (*n* = 11) and those that were not (*n* = 5). Those eligible received VCD induction therapy, whereas the remainder received conventional therapy. The procoagulant activity was measured by means of thrombin generation represented as ETP, peak height, velocity index, lag time, and time-to-peak. PPL activity was measured before and after treatment as PPL clotting time. The red dots and error bars represent the means ± standard deviation, and the black lines show the development from diagnosis to posttreatment of the individual patients. *P<0.05; **P<0.01.
(DOCX)

S1 Table Characteristics of the MM patients at diagnosis and posttreatment in groups with or without HDCT. Of the 16 MM patients, five were eligible for HDCT and received a VCD induction therapy, whereas the remaining 11 received were ineligible for HDCT and thus received conventional therapy. Data are represented as the means ± standard deviation. INR = international normalized ratio; APTT = activated partial thromboplastin time; GFR = glomerular filtration rate; CRP = C-reactive protein; ALAT = alanine transaminase.
(DOCX)
