# Supplementary material for: Extracellular vesicle-associated procoagulant phospholipid and tissue factor activity in multiple myeloma
Source: PLoS One. 2019 Jan 14;14(1):e0210835. doi: 10.1371/journal.pone.0210835 (PMC6331130; doi:10.1371/journal.pone.0210835)
Supplement: S1 Fig — P-values or non-significant (NS) correlations are depicted in the corresponding colour for 20K or 100K pellets. (DOCX) [file pone.0210835.s001.docx]

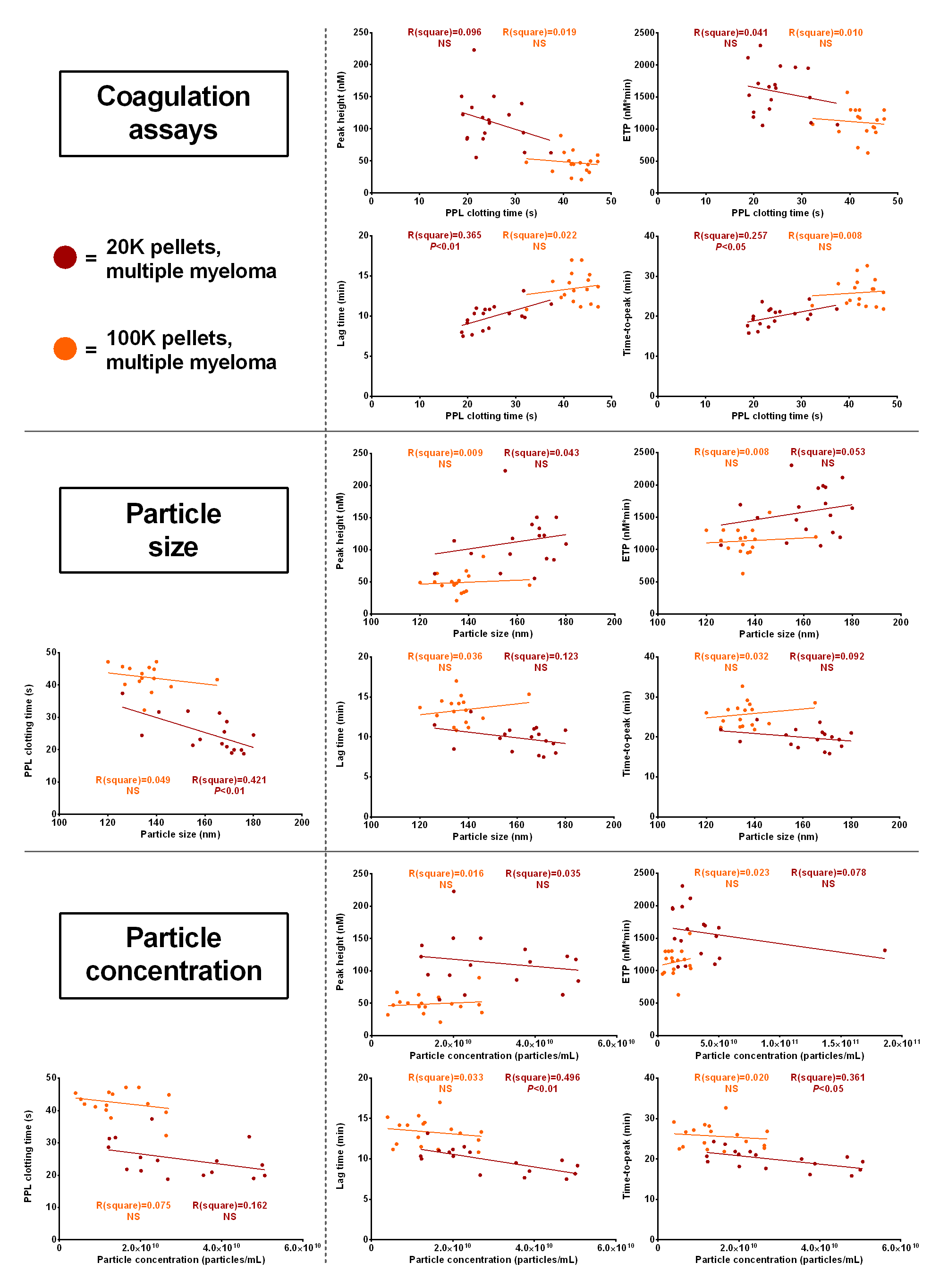


**S1 Fig.** Correlations between coagulation assays and nanoparticle tracking analysis performed on EVs from patients with MM. P-values or non-significant (NS) correlations are depicted in the corresponding colour for 20K or 100K pellets.
