# Supplementary material for: Extracellular vesicle-associated procoagulant phospholipid and tissue factor activity in multiple myeloma
Source: PLoS One. 2019 Jan 14;14(1):e0210835. doi: 10.1371/journal.pone.0210835 (PMC6331130; doi:10.1371/journal.pone.0210835)
Supplement: S2 Fig — Those eligible received VCD induction therapy, whereas the remainder received conventional therapy. The procoagulant activity was measured by means of thrombin generation represented as ETP, peak height, velocity index, lag time, and time-to-peak. PPL activity was measured before and after treatment as PPL clotting time. The red dots and error bars represent the means ± standard deviation, and the black lines show the development from diagnosis to posttreatment of the individual patients. *P<0.05; **P<0.01. (DOCX) [file pone.0210835.s002.docx]

**
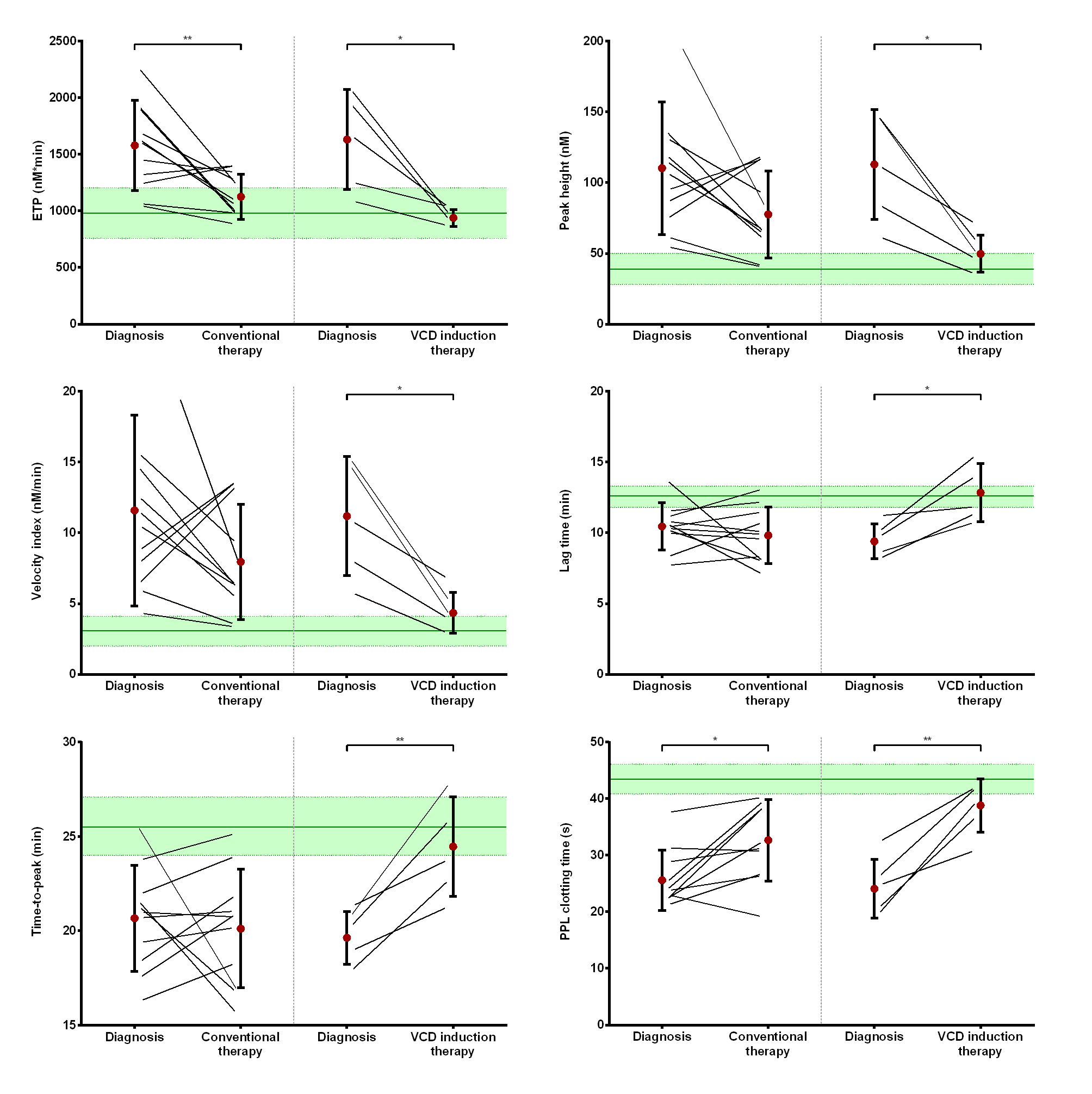
**

**S2 Fig.** The effect of treatment on procoagulant EVs in 20K pellets from MM patients eligible for HDCT (*n*=11) and those that were not (*n*=5). Those eligible received VCD induction therapy, whereas the remainder received conventional therapy. The procoagulant activity was measured by means of thrombin generation represented as ETP, peak height, velocity index, lag time, and time-to-peak. PPL activity was measured before and after treatment as PPL clotting time. The red dots and error bars represent the means ± standard deviation, and the black lines show the development from diagnosis to posttreatment of the individual patients. **P*<0.05; ***P*<0.01.
