# Supplementary material for: Extracellular vesicle-associated procoagulant phospholipid and tissue factor activity in multiple myeloma
Source: PLoS One. 2019 Jan 14;14(1):e0210835. doi: 10.1371/journal.pone.0210835 (PMC6331130; doi:10.1371/journal.pone.0210835)
Supplement: S1 Table — Of the 16 MM patients, five were eligible for HDCT and received a VCD induction therapy, whereas the remaining 11 received were ineligible for HDCT and thus received conventional therapy. Data are represented as the means ± standard deviation. INR = international normalized ratio; APTT = activated partial thromboplastin time; GFR = glomerular filtration rate; CRP = C-reactive protein; ALAT = alanine transaminase. (DOCX) [file pone.0210835.s003.docx]

|  | **Conventional therapy** | | **VCD induction therapy** | |  |
| --- | --- | --- | --- | --- | --- |
|  | **Diagnosis** | **Posttreatment** | **Diagnosis** | **Posttreatment** | *Reference range*  *(male / female)* |
| Number of patients | 11 | - | 5 | - |  |
| INR | 1.1 ± 0.1 | 1.0 ± 0.1 | 1.3 ± 0.2 | 1.0 ± 0.1 | *<1.3* |
| APTT, s | 30 ± 3 | 28 ± 4 | 30 ± 5 | 33 ± 6 | *25-40* |
| Fibrinogen, µmol/L | 10.2 ± 4.1 | 12.0 ± 4.1 | 8.7 ± 1.7 | 15.0 ± 1.9 | *5.0-12.0* |
| D-dimer, mg/L | 0.43 ± 0.56 | 0.29 ± 0.18 | 0.32 ± 0.16 | 0.29 ± 0.09 | *<0.30* |
| Antithrombin, ×E9 IU/L | 0.86 ± 0.15 | 1.00 ± 0.16 | 0.91 ± 0.14 | 1.10 ± 0.13 | *0.85-1.30* |
| Creatinine, µmol/L | 97 ± 27 /  64 ± 11 | 99 ± 34 /  55 ± 15 | 71 ± 9 /  106 ± 7 | 59 ± 2 /  89 ± 26 | *60-105 /*  *45-90* |
| Carbamide, mmol/L | 7.8 ± 3.2 /  6.2 ± 1.1 | 6.7 ± 1.9 /  6.8 ± 1.5 | 6.4 ± 0.5 /  6.0 ± 1.4 | 4.3 ± 1.4 /  5.0 ± 1.1 | *3.5-8.1 /*  *3.1-7.9* |
| Pt-estimated GFR, mL/min | 74 ± 16 | 75 ± 18 | 65 ± 20 | 72 ± 20 | *>60* |
| M-protein, g/L | 42.1 ± 10.2 | 16.6 ± 11.1 | 43.8 ± 20.4 | 5.7 ± 3.2 |  |
| κ-chain, free, mg/L | 308.3 ± 441.2 | 129.2 ± 209.6 | 3901.7 ± 6702.6 | 28.7 ± 20.3 | *3.3-19.4* |
| λ-chain, free, mg/L | 401.8 ± 885.0 | 118.7 ± 354.3 | 56.4 ± 103.8 | 8.2 ± 5.2 | *5.7-26.3* |
| Calcium, mmol/L | 2.43 ± 0.06 | 2.35 ± 0.07 | 2.61 ± 0.11 | 2.44 ± 0.04 | *2.20-2.55* |
| CRP, mg/L | 11.6 ± 29.9 | 9.6 ± 9.4 | 2.0 ± 2.0 | 24.2 ± 34.7 | *<8.0* |
| Albumin, g/L | 29 ± 4 | 33 ± 4 | 30 ± 3 | 32 ± 1 | *34-45* |
| Protein, g/L | 105 ± 11 | 77 ± 11 | 112 ± 14 | 68 ± 6 | *62-78* |
| ALAT, U/L | 20 ± 8 | 18 ± 5 | 32 ± 12 | 25 ± 7 | *10-50* |
| Haemoglobin, mmol/L | 6.1 ± 0.6 /  6.2 ± 0.6 | 6.5 ± 1.1 /  6.6 ± 1.0 | 7.6 ± 1.6 /  5.8 ± 0.4 | 6.7 ± 0.4 /  6.5 ± 0.7 | *8.3-10.5 /*  *7.3-9.5* |
| Erythrocytes, ×E12/L | 3.02 ± 0.28 /  3.37 ± 0.36 | 3.22 ± 0.59 /  3.35 ± 0.66 | 3.78 ± 0.67 /  2.94 ± 0.29 | 3.59 ± 0.21 /  3.27 ± 0.40 | *4.30-5.70 /*  *3.90-5.20* |
| Platelets, ×E9/L | 204 ± 63 /  258 ± 61 | 189 ± 32 /  251 ± 38 | 139 ± 8 /  244 ± 36 | 279 ± 55 /  239 ± 33 | *145-350 /*  *165-400* |
| Leukocytes, ×E9/L | 6.1 ± 2.4 | 5.1 ± 1.2 | 6.9 ± 2.6 | 5.4 ± 1.7 | *3.5-10.0* |

**S1 Table.** Characteristics of the MM patients at diagnosis and posttreatment in groups with or without HDCT. Of the 16 MM patients, five were eligible for HDCT and received a VCD induction therapy, whereas the remaining 11 received were ineligible for HDCT and thus received conventional therapy. Data are represented as the means ± standard deviation. INR = international normalized ratio; APTT = activated partial thromboplastin time; GFR = glomerular filtration rate; CRP = C-reactive protein; ALAT = alanine transaminase.
